# Electronic problem list documentation of chronic kidney disease and quality of care

**DOI:** 10.1186/1471-2369-15-70

**Published:** 2014-05-04

**Authors:** Lipika Samal, Jeffrey A Linder, David W Bates, Adam Wright

**Affiliations:** 1 Division of General Internal Medicine and Primary Care, Brigham and Women’s Hospital, 1620 Tremont St., Suite OBC-03-02 V, Boston, MA 02120, USA; 2 Harvard Medical School, Boston, MA, USA; 3 Harvard School of Public Health, Boston, MA, USA

**Keywords:** Electronic health record, Electronic problem list, Chronic kidney disease, Primary care, Electronic medical record

## Abstract

**Background:**

Chronic kidney disease (CKD) is increasingly common and under-recognized in primary care clinics, leading to low rates of stage-appropriate monitoring and treatment. Our objective was to determine whether electronic problem list documentation of CKD is associated with monitoring and treatment.

**Methods:**

This is a cross-sectional observational study of patients with stage 3 or 4 CKD, defined as two past estimated glomerular filtration rates (eGFR) 15-60 mL/min/1.73 m^2^ separated by 90 days and collected between 2007-2008. We examined the association of problem list documentation with: 1) serum eGFR monitoring test, 2) urine protein or albumin monitoring test, 3) an angiotensin converting enzyme inhibitor or angiotensin receptor blocker (ACE/ARB) prescription, 4) mean systolic blood pressure (BP), and 5) BP control.

**Results:**

Out of 3,149 patients with stage 3 or 4 CKD, only 16% of patients had CKD documented on the problem list. After adjustment for eGFR, gender, and race/ethnicity and after clustering by physician, problem list documentation of CKD was associated with serum eGFR testing (97% with problem list documentation vs. 94% without problem list documentation, p = 0.02) and urine protein testing (47% with problem list documentation vs. 40% without problem list documentation, p = 0.04). After adjustment, problem list documentation was not associated with ACE/ARB prescription, mean systolic BP, or BP control.

**Conclusions:**

Documentation of CKD on the electronic problem list is rare. Patients with CKD documentation have better stage-appropriate monitoring of the disease, but do not have higher rates of blood pressure treatment or better blood pressure control. Interventions aimed at increasing documentation of CKD on the problem list may improve stage-appropriate monitoring, but may not improve clinical outcomes.

## Background

Chronic kidney disease (CKD) is prevalent in the United States and successful approaches to treating the disease could reduce healthcare costs and save lives. Ninety-five percent of the 26 million Americans with CKD are in the early stages of disease and are cared for in primary care clinics by primary care physicians (PCPs) [1]. Nationally accepted guidelines for CKD care describe several aspects of stage-appropriate monitoring and treatment that can delay progression to end stage renal disease (ESRD). Importantly, patients with albuminuria benefit from angiotensin converting enzyme inhibitors (ACE) or angiotensin receptor blockers (ARB) [2-5]. Blood pressure (BP) control delays progression of CKD and prevents mortality from common cardiovascular causes [6,7]. The current state of primary care management of early CKD is largely unknown, though there have been reports of suboptimal management and low adoption of CKD guidelines [8-10]. We do know that recognition of CKD is low [8,11-14]. A systematic review revealed a wide variation of CKD documentation in patient records; the authors compared CKD documentation to a gold standard of estimated glomerular filtration rate and found sensitivities ranged from 0.11-0.45 in the included studies [15]. We do not know whether CKD documentation, and specifically documentation in an electronic problem list, improves the quality of monitoring and treatment.

The electronic problem list in an electronic health record (EHR, also known as EMR) is a specialized tool which has been proven to improve the quality of care for chronic conditions [16-18]. Seeing CKD on the problem list can remind the PCP to order appropriate tests and medications, alert covering physicians to the condition, and trigger clinical decision support programs. For these reasons, federal regulations on Meaningful Use incentives require use of an electronic problem list. We sought to describe the association between problem list documentation of CKD and quality of care for CKD.

## Methods

### Study population and setting

We first gathered data on all of the patients who visited a primary care provider (PCP) at one of 12 primary care clinics in the Brigham and Women’s Primary Care Practice Based Research Network. These clinics serve a diverse set of patients and include hospital-based and community-based clinics, two of which are federally qualified health centers. We included adult patients with stage 3 or 4 CKD, defined as two past estimated glomerular filtration rates (eGFR) between 15-60 mL/min/1.73 m^2^, separated by 90 days and collected during routine clinical care between January 1, 2007 to December 31, 2008 [19]. Our laboratory routinely reports eGFR calculated by the MDRD equation for all serum creatinine measurements. Due to the low rate of testing for urine protein in our population (see Results) we did not include patients with proteinuria who had preserved glomerular filtration rate. We limited the population to patients who had at least one visit with a PCP at one of the practices during 2009. The Partners Institutional Review Board approved this study and granted waiver of consent.

### Electronic health record

The same EHR is used by all 12 clinics. The EHR is a homegrown system called the Longitudinal Medical Record which has been in use since July 2000 [20]. This electronic record allows physicians to maintain problem lists, medication lists, and allergy lists, view laboratory results, enter notes and write electronic prescriptions. Though specialists can add diagnoses to the problem list, our prior work suggests that PCPs are currently most likely to add diagnoses to the problem list [21]. When a provider types in a term that is related to a renal disease, the EHR problem list module presents a list of 15 renal diagnoses (e.g., kidney stones, polycystic kidney disease, and renal cancer) that may be entered as structured data. We obtained problem list entries through a computerized search of electronic patient records for the two codes which indicate CKD (Additional file 1, Longitudinal Medical Record codes 489 and 542). We validated this method with a manual chart review (Additional file 1). At the time of this study there was no clinical decision support to increase documentation of CKD on the problem list [22]. There was a reminder for ACE/ARB prescription for patients with CKD on the problem list and two BP measurements > 140/90 mmHg, and another reminder prompted ACE/ARB prescription for patients with serum creatinine >2.0 and two BP measurements >130/80 mmHg.

### Data analysis

We measured the proportion of patients who had CKD documented on the EHR problem list. We also determined the proportion of patients who had other renal diagnoses on the problem list. We examined the association of potential confounders of the relationship between problem list documentation and quality of care using the chi-squared test. We included demographic characteristics (age, gender, self-reported race/ethnicity) and we accounted for CKD severity as a continuous variable by averaging two consecutive eGFR assessments 90 days apart. We could not account for PCP demographic characteristics, but we examined PCP panels to determine the clustering pattern of patients within panels.

We used similar methods to extract data on the five outcomes of interest, while limiting the search to calendar year 2009. We assessed the association of problem list documentation with five stage-appropriate outcomes, taken from nationally accepted guidelines: 1) serum eGFR test (a measurement during the year 2009, in addition to the two measurements from 2007-2008 used to identify the CKD population), 2) urine albumin/protein test, 3) an ACE or ARB prescription, 4) mean systolic BP, and 5) BP control [6,19]. We accepted several urine protein tests: urine total protein, microalbumin, and microalbumin to creatinine ratio. We used medication list data for ACE/ARB prescription. We used the last recorded blood pressure for blood pressure outcomes and we examined two definitions of BP control: 130/80 mmHg and 140/90 mmHg. These outcomes were chosen due to the strength of evidence for stage-appropriate monitoring and treatment in stages 3 and 4 CKD, and therefore are considered “quality of care” outcomes in the analysis. Due to the fact that PCPs may not check urine albumin in patients who are already on an ACE/ARB, we report the rate of testing in patients who are not on an ACE/ARB. We also performed a subgroup analysis of all outcomes on patients with diabetes listed on the problem list for several reasons: 1) there is evidence that ACE inhibitor therapy benefits CKD patients with proteinuria, but not necessarily CKD patients without proteinuria and 2) there is good evidence that ACE/ARB therapy prevents CKD progression in diabetes [23]. Referral to a nephrologist was not examined in a subgroup analysis due to incomplete data on specialist referrals (Additional file 1).

We used multivariable logistic and linear regression to adjust for confounders and to produce weighted estimates (using the LSMEANS feature of the GENMOD procedure in SAS, SAS Institute, Cary, NC). We first analyzed the data assuming that each event was independent. Then we conducted analyses accounting for clustering by PCP and found similar results. All unadjusted and adjusted estimates are presented after accounting for clustering by PCP.

## Results

### Patient characteristics

Of the 79,605 patients who made a visit to a PCP in 2009, there were 3,149 patients with stage 3 or 4 CKD as defined by two eGFR results between 15-60 mL/min/1.73 m^2^ separated by 90 days. Patients had a mean age of 74 years (range 26-104 years) and were predominantly female (Table 1). The mean eGFR was 46 mL/min/1.73 m^2^ (standard deviation ±10 mL/min/1.73 m^2^). Race/ethnicity rates were 67% White, 23% Black, 8% Hispanic, and 1.5% Asian.

**Table 1:** Characteristics of stage 3 and 4 CKD patients in a network of primary care practices and association with CKD documentation

	**Total**	**CKD on problem list**	**CKD not on problem list**	**P value**
	**(N = 3149)**	**(N = 488)**	**(N = 2661)**	
Patient characteristics				
Age, mean (SD)	73.6 (12.1)	73.9 (12.7)	73.6 (11.9)	P = 0.62
Female gender, N (%)	223 (64%)	229 (47%)	1809 (68%)	P < 0.0001
eGFR, mean (SD)	45.9 (9.98)	37.6 (10.1)	47.5 (9.2)	P < 0.0001
Race/Ethnicity, N (%)				P = 0.004
White	2064 (67%)	288 (60%)	1776 (68%)	
Black	723 (23%)	138 (29%)	585 (22%)	
Hispanic	260 (8.4%)	49 (10%)	211 (8%)	
Asian	45 (1.5%)	8 (1.7%)	37 (1.5%)	
Diabetes	866 (28%)	196 (40%)	670 (25%)	
Hypertension	2118 (67%)	384 (79%)	1734 (65%)	

### Physician panel characteristics

Of the patients with a laboratory diagnosis of stage 3 or 4 CKD, 3,074 patients were assigned to one of the 269 PCPs within the Brigham and Women’s Primary Care Practice Based Research Network. Each PCP panel included 1 to 173 patients. We treated the 75 patients who were not assigned to a PCP as independent clusters.

### Problem list entry of chronic kidney disease

Out of 3,149 patients with laboratory evidence of stage 3 or 4 CKD, 488 patients (16%) had CKD on the problem list. Problem list documentation was more likely in patients with lower eGFR, male gender, Black race/ethnicity, or Hispanic race/ethnicity (Table 1). In terms of PCP panels, the PCPs had between 1 and 16 patients with CKD documented on the problem list (out of a total of 1 to 173 patients with laboratory evidence of stage 3 or 4 CKD). An additional 7% of patients with laboratory evidence of stage 3 or 4 CKD had one of the other renal diagnoses on the problem list (see Additional file 1).

### Quality of care outcomes

We then sought to determine whether CKD documentation correlated with five quality of care outcomes during calendar year 2009: 1) serum eGFR test, 2) urine protein or albumin test, 3) an angiotensin converting enzyme inhibitor or angiotensin receptor blocker prescription, 4) mean systolic blood pressure, and 5) blood pressure control (separate analyses for goal <130/80 mmHg and <140/90 mmHg). The percentage of patients with a serum eGFR test during the one year study period was 94% and there was an association with CKD documentation after adjusting for clustering by PCP (CKD documentation 98% vs. no CKD documentation 93%, p < 0.0001). A model adjusting for eGFR, gender, and race/ethnicity, showed a significant association as well (97% vs. 94%; p = 0.02; Table 2). The overall rate of urine protein or albumin testing was 40% and there was a significant association with problem list documentation (61% vs. 41%, p <0.0001), which persisted after adjustment for eGFR, gender, and race (47% vs. 40%, p = 0.04; Table 2). The overall rate of ACE/ARB prescription was 65% and there was a significant association with problem list documentation (75% vs. 66%, p <0.0001), but the association was no longer significant after adjustment for eGFR, gender, and race (Table 2). The rate of urine protein or albumin testing in patients who were not on an ACE/ARB was lower overall (25%) and there was a significant association with problem list documentation (48% vs. 22%, p < 0.0001). The mean BP of patients was 133/72 mmHg. There were no significant associations between problem list documentation and mean systolic BP or mean diastolic BP before or after adjustment (Table 2). With a BP goal of 140/90 mmHg, 71% were under control and the likelihood of being under control was not associated with problem list documentation (Table 2). With a blood pressure goal of 130/80 mmHg, 45% were under control and the likelihood of being under control was not associated with problem list documentation (Table 2).

**Table 2:** Associations of CKD documentation with monitoring and treatment

		**Unadjusted estimates**^**a**^			**Adjusted estimates**^**ab**^		
	**Total (N = 2965)**	**CKD on problem list (N = 488)**	**CKD not on problem list (N = 2661)**	**p value**	**CKD on problem list (N = 483)**	**CKD not on problem list (N = 2623)**	**p value**
Serum eGFR, %	94%	98%	93%	P < 0.0001	97%	94%	P = 0.002
Urine protein, %	40%	61%	41%	P < 0.0001	47%	40%	P = 0.04
ACE/ARB, %	65%	75%	66%	P < 0.0001	68%	67%	P = 0.62
Systolic BP, mean	132.5 mmHg	132.4 mmHg	132.5 mmHg	P = 0.88	131.9 mmHg	132.1 mmHg	P = 0.84
Diastolic BP, mean	72.0 mmHg	71.2 mmHg	72.1 mmHg	P = 0.12	72.3 mmHg	71.9 mmHg	P = 0.50
BP <140/90 mmHg	71%	69%	72%	P = 0.22	72%	72%	P = 0.99
BP <130/80 mmHg	45%	46%	45%	P = 0.74	46%	46%	P = 0.86

^a^Adjusted for clustering by physician.

^b^Weighted estimates after adjustment for eGFR, gender, race.

In the subgroup of 866 patients with diabetes, the percentage of patients with a serum eGFR test during the one year study period was 96% and there was an association with CKD documentation (after adjusting for clustering by PCP; CKD documentation 99% vs. no CKD documentation 96%, p = 0.05). However, the association was no longer significant after adjustment for eGFR, gender, and race. The rate of urine protein or albumin testing in patients with diabetes was 77% and there was no significant association with CKD problem list documentation. This is compared to a rate of 26% in the 2283 patients without diabetes, where there is a significant association of urine protein or albumin testing with CKD documentation (44% vs. 24%, p < 0.0001), but the association was no longer significant after adjustment for eGFR, gender, and race. The rate of ACE/ARB prescription in patients with diabetes was 86% and there was no significant association with CKD problem list documentation. There were no significant associations between CKD documentation and blood pressure outcomes within the diabetes subgroup.

## Discussion

We found that one out of every six patients with laboratory evidence of stage 3 or 4 CKD had CKD documented as a diagnosis on the problem list. The low documentation of this chronic illness shows that there are opportunities to improve the quality of care for CKD patients. Documentation was associated with certain patient characteristics such as disease severity, suggesting that there is a severity threshold above which PCPs are more likely to document CKD on the problem list.

We also found that demographic characteristics (i.e., male gender, Black race, and Hispanic ethnicity) are associated with CKD documentation, which is consistent with prior studies. One possibility is that CKD documentation is more common in groups that have higher serum creatinine levels for a given eGFR (men and Black patients [24]); this fact may suggest that physicians are still using the serum creatinine level to diagnose CKD. However, patients of Hispanic ethnicity typically have lower creatinine levels than white patients [25]. Therefore, the relatively higher rate of CKD documentation in Hispanic patients would not be related to creatinine level.

Encouragingly, patients with CKD on the EHR problem list were more likely to receive stage-appropriate monitoring including serum eGFR testing and urine protein testing. The higher rate of urine protein testing is important because recent evidence and expert opinion support the idea that urine protein testing in addition to serum creatinine-based measures provide superior risk stratification [26,27]. When we examined urine protein testing in the diabetes subgroup, we did not see an association with CKD documentation, which may be due to competing demands in patients with multiple comorbidities [28].

We did not find a significant relationship between CKD documentation and higher quality treatment, specifically ACE/ARB treatment, after adjustment for age, race and eGFR. Finally, the mean systolic and diastolic BP values were not associated with CKD documentation and when we examined BP control as a dichotomous outcome we found that CKD documentation was not associated with the likelihood of blood pressure control.

The low rate of documentation is in agreement with studies that examined CKD documentation using billing codes rather than the EHR problem list. For example, a study which examined billing codes found that CKD was included as a billing code only 14% of the time [14]. In a study conducted within an integrated delivery system CKD documentation was 24% and the investigators found that male gender and Black race were significantly associated with documentation, just as we did [29]. Another study reported a combined electronic problem list and billing diagnosis rate of 19% [30]. Interestingly, another study examined clinic notes for patients with low eGFR and reported a 70% rate of documentation in the text of the clinic notes, suggesting that our study examining the electronic problem list falls prey to the same limitations as research using billing codes; neither one reflects true PCP awareness of CKD [12]. However, we still support observation and intervention to improve the problem list because documentation in the free text of clinical notes is not as useful for quality measurement, health services research, or clinical decision support interventions, and potentially is a barrier to team-based primary care models, such as the patient-centered medical home.

The strength of this study of electronic problem list documentation was the large dataset from the clinical information system of a primary care network serving a diverse population. Despite these strengths, our study has several limitations. Though we utilized longitudinal data, we analyzed the data in a cross-sectional fashion. Therefore, we cannot assume that associations are causal in nature. PCPs may choose not to add CKD as a separate diagnosis when a patient already has a renal diagnosis (e.g., polycystic kidneys). It is possible that certain PCPs characteristics (e.g., thoroughness, visit duration, practice style) confound the association between documentation and quality of care, but we chose to cluster patients within PCP panels for this reason. When considering limitations of measures, data on laboratory tests was limited to those performed in our hospital’s affiliated laboratories, but the population only included patients who had seen one our affiliated PCPs during the study period and all lab orders are automatically routed through our central laboratory. We attempted to control for severity of CKD by adjusting for eGFR as a continuous measure, but this may not accurately reflect severity of disease. Similarly, we only captured ACE/ARB prescriptions written using our electronic prescribing module, but all PCPs were using this module almost exclusively during the study period. We did not include serum creatinine tests unless an eGFR was reported by the lab as well, but eGFR has been routinely reported by our lab for the past decade. We used the last BP reading rather than a synthesis of multiple blood pressure readings over the study period, which may affect the validity of the measure [31]. Due to the limitations of the dataset, we do not know how severe the albuminuria was for each patient. In terms of ACE/ARB prescription and blood pressure control, the overlap between management of CKD and management of both diabetes and hypertension limits our ability to distinguish any impact of CKD documentation from interventions affecting diabetes and hypertension management. We were unable to include outcomes that would be specific to CKD, such as dose adjustment of nephrotoxic medications. These limitations were introduced by the method of data collection which relied on electronic data collected during routine care, which is a low-cost, feasible approach to health services research, and we have verified the validity of the data with a manual chart review (Additional file 1).

## Conclusion

Documentation of CKD on the electronic problem list was uncommon and more likely in patients with certain demographic characteristics and more severe CKD. CKD documentation was positively associated with stage-appropriate monitoring, both serum eGFR testing and urine protein testing. However, CKD documentation was not associated with higher rates of blood pressure treatment or better blood pressure control. Therefore, interventions aimed at increasing documentation of CKD can improve monitoring, but documentation alone is not sufficient to improve treatment or intermediate clinical outcomes.

## Competing interests

The authors declare that they have no competing interests.

## Authors’ contributions

LS had complete access to data, performed the statistical analysis, and drafted the manuscript. JAL, DWB, and AW have each made substantial contributions to conception and design, and interpretation of data; have been involved in revising the manuscript; and have given final approval of the version to be published. All authors read and approved the final manuscript.

## Pre-publication history

The pre-publication history for this paper can be accessed here:

http://www.biomedcentral.com/1471-2369/15/70/prepub

## Supplementary Material

Additional file 1 **A. Options provided within the electronic medical record to add renal diagnoses to the problem list.** B. Data Validation study. Click here for file
