# Supplementary material for: Electronic problem list documentation of chronic kidney disease and quality of care
Source: BMC Nephrol. 2014 May 4;15:70. doi: 10.1186/1471-2369-15-70 (PMC4021481; doi:10.1186/1471-2369-15-70)
Supplement: Additional file 1 — A. Options provided within the electronic medical record to add renal diagnoses to the problem list. B. Data Validation study. [file 1471-2369-15-70-S1.docx]

Additional file 1

**A. Options provided within the electronic medical record to add renal diagnoses to the problem list**

| Electronic Problem List Code | Problem Description |
| --- | --- |
| 489 | Chronic renal dysfunction |
| 542 | Renal insufficiency |
| 135 | End stage renal disease |
| 706 | Early renal disease |
| 707 | Hemodialysis |
| 708 | Peritoneal dialysis |
| 818 | Nephropathy |
| 1012 | Kidney transplant |
| 212 | Kidney stone |
| 335 | Polycystic kidneys |
| 371 | Renal cancer |
| 490 | Acute renal failure |
| 605 | Glomerulonephritis |
| 1070 | Interstitial nephritis |
| 1409 | Horseshoe kidney |

**B. Data Validation study**

To assess data quality we randomly selected 100 patients with eGFR <60 mL/min/1.73 m^2^ from our study population and performed manual chart review. When checking the inclusion criteria we found that 100% of patients had two past eGFR <60 mL/min/1.73 m^2^ separated by 90 days collected over the period 1/1/2007-12/31/2008 and 97% of patients had a primary care visit during 2009. We did not have access to mortality data, but 93% of patients had notes extending past December 2009. Demographic variables (age, race, gender) were confirmed in 98% of cases. When we reviewed data on referral to a nephrologist we determined that our search algorithm returned a false negative 5% of the time and that any patient who was seen by a nephrologist as an inpatient was counted as a false positive.

The exposure variable was correctly identified in 97% of cases; in three cases the automated search did not identify a coded entry on the problem list. We also found one case where a free text entry documented CKD, but was not identified by the automated search. The outcome measures were 98%-100% correct: 1) the automated search for eGFR measurement was 100% accurate, 2) the automated search for urine protein tests identified 98%, 3) the automated search erroneously counted one ACE/ARB prescription that was not present on manual review of the medication list, and 4) the automated search identified 98% of systolic blood pressure readings. We present this data as an aid to others using EHR data for health services or epidemiologic research.
